# Identification of Brugada syndrome based on P-wave features: an artificial intelligence-based approach

**DOI:** 10.1093/europace/euad334

**Published:** 2023-11-07

**Authors:** Beatrice Zanchi, Francesca Dalia Faraci, Ali Gharaviri, Marco Bergonti, Tomas Monga, Angelo Auricchio, Giulio Conte

**Affiliations:** Department of Innovative Technologies, Institute of Digital Technologies for Personalized Healthcare of SUPSI, Lugano, Switzerland; Department of Quantitative Biomedicine, University of Zurich, Zurich, Switzerland; Department of Innovative Technologies, Institute of Digital Technologies for Personalized Healthcare of SUPSI, Lugano, Switzerland; Center for Computational Medicine in Cardiology, USI, via La Santa 1, 6900, Lugano, Switzerland; Centre of Cardiovascular Science, University of Edinburgh, Edinburgh, Scotland; Division of Cardiology, Cardiocentro Ticino Institute, Ente Ospedaliero Cantonale, via Tesserete 64, 6900, Lugano, Switzerland; Faculty of Biomedical Sciences, Università della Svizzera Italiana (USI), Lugano, Switzerland; Center for Computational Medicine in Cardiology, USI, via La Santa 1, 6900, Lugano, Switzerland; Division of Cardiology, Cardiocentro Ticino Institute, Ente Ospedaliero Cantonale, via Tesserete 64, 6900, Lugano, Switzerland; Faculty of Biomedical Sciences, Università della Svizzera Italiana (USI), Lugano, Switzerland; Center for Computational Medicine in Cardiology, USI, via La Santa 1, 6900, Lugano, Switzerland; Division of Cardiology, Cardiocentro Ticino Institute, Ente Ospedaliero Cantonale, via Tesserete 64, 6900, Lugano, Switzerland; Faculty of Biomedical Sciences, Università della Svizzera Italiana (USI), Lugano, Switzerland

**Keywords:** Brugada syndrome, P-wave, Artificial intelligence, Machine learning, Electrocardiogram

## Abstract

**Aims:**

Brugada syndrome (BrS) is an inherited disease associated with an increased risk of ventricular arrhythmias. Recent studies have reported the presence of an altered atrial phenotype characterized by abnormal P-wave parameters. The aim of this study was to identify BrS based exclusively on P-wave features through an artificial intelligence (AI)-based model.

**Methods and results:**

Continuous 5 min 12-lead ECG recordings were obtained in sinus rhythm from (i) patients with spontaneous or ajmaline-induced BrS and no history of AF and (ii) subjects with suspected BrS and negative ajmaline challenge. The recorded ECG signals were processed and divided into epochs of 15 s each. Within these epochs, P-waves were first identified and then averaged. From the averaged P-waves, a total of 67 different features considered relevant to the classification task were extracted. These features were then used to train nine different AI-based supervised classifiers. A total of 2228 averaged P-wave observations, resulting from the analysis of 33 420 P-waves, were obtained from 123 patients (79 BrS+ and 44 BrS−). Averaged P-waves were divided using a patient-wise split, allocating 80% for training and 20% for testing, ensuring data integrity and reducing biases in AI-based model training. The BrS+ patients presented with longer P-wave duration (136 ms vs. 124 ms, *P* < 0.001) and higher terminal force in lead V1 (2.5 au vs. 1.7 au, *P* < 0.01) compared with BrS− subjects. Among classifiers, AdaBoost model had the highest values of performance for all the considered metrics, reaching an accuracy of over 81% (sensitivity 86%, specificity 73%).

**Conclusion:**

An AI machine-learning model is able to identify patients with BrS based only on P-wave characteristics. These findings confirm the presence of an atrial hallmark and open new horizons for AI-guided BrS diagnosis.

## Graphical Abstract

**Graphical Abstract:**
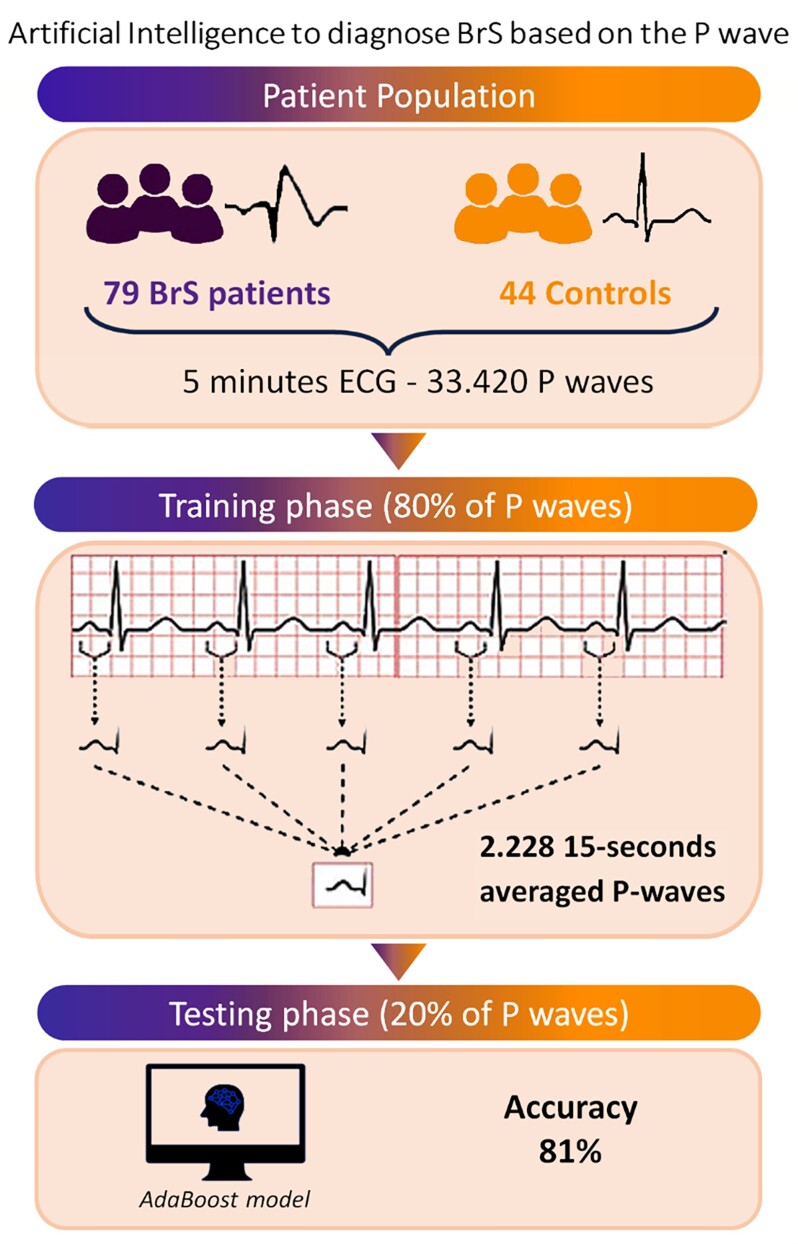

**What’s new?**

- Patients with Brugada syndrome (BrS) present with a specific ECG phenotype characterized by P-waves abnormalities.
- An artificial intelligence (AI) machine-learning model is able to identify patients with BrS based only on P-wave features.
- These findings confirm the presence of an atrial hallmark and open new horizons for AI-guided BrS diagnosis.

## Introduction

Brugada syndrome (BrS) is an inherited arrhythmia syndrome diagnosed in patients with no overt heart disease in the presence on the 12-lead electrocardiogram (ECG) of a specific ventricular phenotype consisting in a coved-type 2 ST-segment elevation of at least 2 mm in one of the right precordial leads.^1,2^ In addition to the risk of ventricular arrhythmias, a considerable portion of patients with BrS are exposed to a risk of atrial arrhythmias, including atrial fibrillation (AF), reflecting the involvement of the atrial myocardium in the manifestation of the syndrome.^3–5^

Recent studies have reported the presence in BrS patients of an altered atrial phenotype characterized by abnormal P-wave parameters and prolonged atrial conduction time at ECG imaging, despite the absence of history of AF, confirming that the arrhythmic substrate is not solely restricted to the ventricular level.^6–8^ These patients present with an atrioventricular ECG phenotypic mismatch, being P-waves abnormalities detected even in the absence of an overt Brugada type 1 ECG.^6^ To date, there are no data on the potential value of assessing P-wave characteristics to diagnose BrS.

Artificial intelligence (AI) applications and machine learning (ML) algorithms have recently gained attention for their ability to recognize patterns associated with cardiovascular diseases, including AF and inherited arrhythmia syndromes.^9–14^ However, no AI-based model to identify BrS patients based on non-ventricular ECG parameters has been reported so far.

The aim of this study was to evaluate P-wave parameters of a series of patients with BrS and healthy subject controls with negative ajmaline challenge and to apply AI methods to identify BrS through the observation of electrical alterations of the P-waves.

## Methods

### Study population

A consecutive series of BrS patients was identified at Cardiocentro Ticino Institute, EOC (Lugano, Switzerland). Institutional and Ethics Committee approval was obtained (Swiss Ethics, approval number: BASEC 2019-00754/CE 3476), and all identified patients gave informed consent to participate in the study.

The diagnosis of BrS was established based on the current guidelines and on the Shanghai Score System criteria.^2^ Electrocardiogram recordings were classified as either coved-type (type 1), saddleback (type 2), or normal. An ECG was considered diagnostic of BrS only if a coved-type ST elevation ≥ 2 mm was documented in ≥1 lead from V1 to V3 in the presence or absence of a sodium channel blocker agent. Medical and family history evaluation, physical examination, bi-dimensional transthoracic echocardiography, cardiac magnetic resonance imaging, and genetic testing were performed in all cases.

Moreover, a control group of healthy individuals referred for suspected BrS who tested negative after ajmaline challenge were included. Ajmaline (1 mg/kg) was administered intravenously over a 5 min period. All baseline and ajmaline challenge ECGs were recorded at a paper speed of 25 mm/s and amplitude of 10 mm/mV with the right precordial leads positioned at the sternal margin of the second, third, and fourth intercostal spaces. Ajmaline challenge was considered negative if no Brugada type 1 ECG was unmasked during the test.

Individuals with previous history of atrial arrhythmias, including AF, cardiac structural abnormalities, or with an age below 18 years were excluded from the study.

### Electrocardiogram acquisition and pre-processing

Brugada syndrome and control group subjects underwent a 12-lead ECG recording obtained in sinus rhythm by a high-resolution ECG machine (CARDIOVIT CS-200 Excellence; Schiller) having a sampling frequency of 1 kHz and a band-pass filter with cut-off frequencies set at 0.5–300 Hz. Each recording lasted from a minimum of 30 s to a maximum of 5 min per patient. Moreover, at the time of ECG recordings, all patients presented with a non-diagnostic ECG (absence of overt Brugada type 1 ECG).

The recordings were automatically filtered using a line frequency filter for the suppression of superimposed 50 or 60 Hz sinusoidal interference through adaptive digital filtering. To remove the remaining noise, the signals were filtered by using a 5th order Chebyshev low-pass filter with a cut-off frequency set at 100 Hz. Similar to previous studies, the extraction of P-wave indexes was done on averaged P-waves.^6,7^ The filtered signal was divided into 15 s epochs, and all the valid P-waves were then identified. At the end of windowing process, multiple 15 s epochs per patient were obtained. Segmentation of original signal into 15 s epochs was performed for two key reasons as follows: (i) to enhance the quality of the averaged P-wave and, consequently, to improve the precision of P-wave features extraction by reducing noise and artefacts; and (ii) to capture the full spectrum of ECG variability, as these signals can exhibit dynamic changes (e.g. heart rate variability). This approach provides a more comprehensive representation of physiological phenomena throughout the recording.

**Figure 1:**
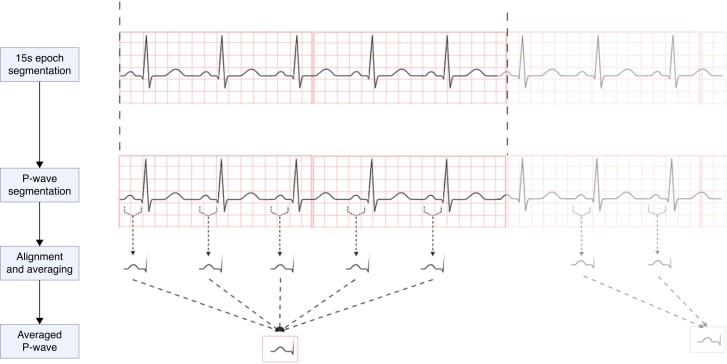
Procedure to obtain averaged P-waves on ECG signal 15 s epoch.

To isolate P-waves in each 15 s epoch, an algorithm similar to that proposed by Pan and Tompkins^15^ was applied to the ECG signals, in order to detect R-waves. This algorithm acts as a high-pass filter, highlighting the high-frequency QRS complexes. The P-waves were then extracted from a 300 ms window starting 350 ms before the R-peak. Ectopic atrial beats or P-waves with excessive noise were eliminated by computing the cross-correlation function between each P-wave and a P-wave template. Each P-wave was tested as a P-wave template and the one that produced the highest average cross-correlation was used as the final P-wave template. Finally, before averaging, P-waves were aligned according to the time lag at which the cross-correlation function between the P-wave template and each individual P-wave reached its maximum. This framework was repeated for each ECG lead. The procedure to obtain averaged P-wave per epoch is shown in *Figure 1*.

### P-wave features extraction

For each averaged P-wave instance, two feature groups were extracted as follows: global and local features. Global features refer to the ones that are averaged between the considered leads, which integrate more information coming from different ECG leads, or that are extracted on a single ECG lead. This group includes five different features as follows: P-wave duration, PR interval, P-wave terminal force in lead V1 (PTFV1), full width at half maximum (FWHM), and P-wave axis. Specifically, P-wave duration and PTFV1 are indicators of left atrial depolarization impairment. Full width at half maximum is computed as a time interval in which the P-wave assumes a value > 50% of its maximum amplitude. It is considered a marker of P-wave amplitude dispersion over time.

Local features refer to those that are singularly extracted from each of the 12 ECG leads. In this group, the following five are included: P-wave area, number of P-wave peaks, maximum P-wave amplitude, P-wave entropy, and P-wave sample entropy. Given that each of these characteristics has been extracted for every ECG lead, the total number of local features amounts to 60. In detail, P-wave area assesses for atrial structure, as it correlates to atrial volume index, as previously shown.^14^ P-wave amplitude represents the maximum electrical force through which the stimulus propagates between the atrial chambers. P-wave entropy represents the amount of uncertainty (e.g. the degree of repeatability) of the signal’s amplitude profile over time. Hence, it is an indicator of the repeatability of certain patterns within the averaged P-wave. Entropy and sample entropy are computed as previously reported.^6,7^ The values of P-wave feature exhibit dependence on the computation methodology and were derived using the absolute value, as reported in the literature.^6^

Considering local, global, and clinical characteristics such as patient’s age and gender, a total of 67 different features were extracted from the averaged P-wave.

### Model training and validation

The original dataset was divided into training and test sets (451 observations, 20% of instances). The term ‘observation’ refers to the set of 67 characteristics derived from a single 15 s averaged P-wave. The initial recording duration ranged from 30 s to 5 min, and all recordings were segmented into 15 s epochs. Consequently, patients with longer signal recording had a more significant impact on both training and testing than patients with shorter recordings. Specifically, a minimum of 2 and a maximum of 20 observations per patient were exploited during the algorithms training.

During splitting, we ensured that all observations associated with each individual patient fell only in the training, or validation, or test set, in order to limit biases. Moreover, after dividing training set and test set, it was ensured that test set contained only age- and sex-matched patients. In the test set, the mean age of Brugada and control subjects was 42 ± 15 years and 38 ± 12 years, respectively (*P* = 0.237). Clinical features of patients included in the test set are shown in Supplementary material online, *Table S1* (Supplementary material).

Supervised ML models were trained to classify BrS patients against negative-ajmaline control group based only on P-wave characteristics. Two sets of classifiers were exploited. Each algorithm was trained to maximize the F1-score metric during the hyperparameters tuning procedure. Basic classifiers included K-Nearest Neighbors (KNN), Support Vector Machine (SVM), and Decision Tree (DT) while ensemble classifiers included Bagging of Decision Trees, Majority Voting, Stacking, Adaptive Boosting (AdaBoost), and Gradient Boosting (GBoost). Models’ hyperparameters tuning was carried out using grid search or random search techniques, depending on search space size. K-fold cross-validation technique (with K = 10 folds) was used to validate classifiers’ performances during the hyperparameters tuning procedure. Different data balancing techniques were tested to improve classifiers’ performances.

### Performance metrics

In order to evaluate and compare classifier performances, overall Accuracy (Acc.), Macro-Averaging F1-score (MF1), Weighted-Averaging F1-score (WF1), sensitivity (Sen.), and specificity (Spec.) were computed.


$$\mathrm{Accuracy}=\;\frac{\mathrm{True}\;\mathrm{Positives}+\mathrm{True}\;\mathrm{Negatives}}{\mathrm{True}\;\mathrm{Positives}+\mathrm{True}\;\mathrm{Negatives}+\mathrm{False}\;\mathrm{Positives}+\mathrm{False}\;\mathrm{Negatives}}$$

$$\mathrm{Sensitivity}=\;\frac{\mathrm{True}\;\mathrm{Positives}}{\mathrm{True}\;\mathrm{Positivies}+\mathrm{False}\;\mathrm{Negatives}}$$

$$\mathrm{Specificity}=\;\frac{\mathrm{True}\;\mathrm{Negatives}}{\mathrm{True}\;\mathrm{Negatives}+\mathrm{False}\;\mathrm{Positives}}$$

$$\mathrm{Precision}=\;\frac{\mathrm{True}\;\mathrm{Positives}}{\mathrm{True}\;\mathrm{Positives}+\mathrm{False}\;\mathrm{Positives}}$$

$$\mathrm{Macro}\;\mathrm{Averaging}\;F1\;\mathrm{Score}\;=2*\;\frac{\mathrm{Precision}*\mathrm{Sensitivity}\;}{\mathrm{Precision}+\mathrm{Sensitivity}}$$

Weighted-averaging F1-score is a weighted average of the class-wise F1 scores, the weights of which are determined by the number of samples available in that class as follows:


$$\mathrm{Weighted}\;\mathrm{Averaging}\;F1\;\mathrm{Score}=\;\overset{N}{\underset{i=1}{\sum }}\;w_{i\;}*F1{\mathrm{Score}}_{i}$$

$$w_{i}=\;\frac{\mathrm{Number}\;\mathrm{of}\;\mathrm{samples}\;\mathrm{in}\;\mathrm{class}\;i}{\mathrm{Total}\;\mathrm{number}\;\mathrm{of}\;\mathrm{samples}}.$$

#### Statistics

Continuous data were presented as mean ± standard deviation and compared with the Mann–Whitney *U* test. Categorical data were expressed as number and percentage of population and compared with the Fisher’s exact test. Statistical analyses were performed with SciPy Python-based library (version 1.10.1). All tests were two-sided, and a *P*-value below 0.05 was considered statistically significant. For the statistical tests performed on averaged P-wave features, Bonferroni adjustment for multiple tests was applied and significant level was set to 0.00076.

## Results

### Study population

A total of 123 subjects (67% male, mean age: 43 ± 15 years) were included. Brugada syndrome was diagnosed in 79 patients, while 44 subjects underwent ajmaline challenge for suspected BrS and drug test was negative in all of them.

Study population characteristics are shown in *Table 1*. Up to 29% of BrS patients presented with a spontaneous type 1 ECG. Echocardiographic parameters with regard to atrial dimensions were within the range of normality in all BrS patients and control subjects.

**Table 1:** Study population characteristics

	Brugada patients (*n* = 79)	Negative Ajmaline subjects (*n* = 44)	*P*-value
Clinical characteristics			
Male sex, *n* (%)	55 (69.6%)	28 (63.6%)	0.64
Age (years)	47 ± 14	36 ± 14	0.03
Family history of SCD, *n* (%)	15 (18.9%)	4 (9.1%)	0.23
Syncope, *n* (%)	15 (18.9%)	8 (18.2%)	0.91
Previous sustained VAs, *n* (%)	7 (8.9%)	—	—
Previous ICD implantation, *n* (%)	16 (20.2%)	—	—
*SCN5A* P/LP variant, *n* (%)	10 (12.6%)	—	—
Heart rate (bpm)	82 ± 12	78 ± 10	0.83
Echocardiographic parameters			
LVEF (%)	60.2 ± 2.1	60.7 ± 2.8	0.91
LA diameter (mm)	31.5 ± 5.7	30.7 ± 6	0.74
LAVI (mL/m^2^)	25.2 ± 5.1	24.8 ± 7.8	0.61
RAVI (mL/m^2^)	22.8 ± 2.7	21.35 ± 6.6	0.18
Brugada ECG pattern, *n* (%)			
Spontaneous Brugada type 1	23 (29.1%)	—	—
Ajmaline-induced Brugada type 1	55 (69.6)%	—	—
Fever-induced Brugada type 1	1 (1.3%)	—	—
Brugada type 2 ECG pattern	25 (31.2%)	11 (25%)	0.56

Data are presented as mean ± standard deviation.

*α* = 0.05. SCD, sudden cardiac death; VAs, ventricular arrhythmias; P/LP, pathogenic/likely pathogenic; LVEF, left ventricular ejection fraction; LA, left atrial; LAVI, Left atrial volum index; RAVI, right atrial volume index; ECG, electrocardiogram; ICD, implantable cardioverter defibrillator.

### P-wave parameters analysis

At the end of pre-processing, a total of 1444 and 784 averaged P-wave observations were obtained for BrS and control group subjects, respectively.

*Table 2* shows P-wave global features of the two groups. BrS subject presented with longer P-wave duration (136 ms vs. 124 ms, *P* = 0.0001). In contrast, PR interval, FWHM, terminal force in V1 (TFV1), and P-wave axis were not significantly different among the two groups.

**Table 2:** P-wave global features

Parameter	Brugada patients	Negative Ajmaline subjects	*P*-value
Duration [ms]	136 (125–145)	124 (113–134)	**0**.**0001**
PR interval [ms]	177 (161–190)	170 (134–181)	0.0644
TFV1 [au]	2.5 (1.2–3.2)	1.7 (0.9–2)	0.0052
FWHM [ms]	57 (42–58)	46 (40–52)	0.0519
Axis [°]	56 (55–77)	60 (51–75)	0.2341

Data are presented as median (25–75 percentiles). Values in bold refer to statically significant values.

FWHM, full width at half maximum; TFV1, terminal force in V1.

*α* = 0.00076.

In *Table 3*, local features analysis results are reported for each lead. Interestingly, the only statistically significant difference was related to P-wave area. More specifically, P-wave area was higher for BrS group in lead V1 (3.3 au vs. 2.3 au, *P* = 0.0006). No statistically significant variations were found in P-wave amplitude across all examined leads. Moreover, entropy, sample entropy, and the number of P-wave peaks did not differ among the two groups.

**Table 3:** P-wave local features

Parameters	ECG leads	Brugada	Negative ajmaline	*P*-value
Area [au]	I	4 (2.7–5)	3.7 (2.9–4.5)	0.7899
	II	10 (8–11.7)	8.6 (5.9–11)	0.0127
	III	6.4 (4–5.4)	5 (2.7–7.3)	0.0158
	aVR	7 (5.8–8)	6 (4.7–7.2)	0.0241
	aVL	2.4 (1.6–3)	2 (1–2.7)	0.0258
	aVF	8 (5.9–9.7)	6.8 (4.3–8.8)	0.0101
	**V1**	**3.3 (2–4.1)**	**2.3 (1.8–2.7)**	**0.0006**
	V2	2.5 (1.7–3.2)	2.4 (1.7–3)	0.9684
	V3	5.3 (4–6.5)	4.7 (3.2–6)	0.0629
	V4	5.4 (4.2–6.4)	4.7 (3–6)	0.0142
	V5	5.2 (3.8–5.9)	4.3 (2.8–5.4)	0.0066
	V6	4.9 (4.6–5.9)	4 (2.5–5)	0.0048
Amplitudine [mV]	I	0.08 (0.06–0.1)	0.08 (0.07–0.1)	0.5923
	II	0.2 (0.16–0.24)	0.19 (0.14–0.22)	0.0888
	III	0.14 (0.1–0.18)	0.12 (0.08–0.16)	0.0311
	aVR	0.14 (0.11–0.16)	0.13 (0.1–0.15)	0.1003
	aVL	0.07 (0.05–0.08)	0.06 (0.04–0.07)	0.0506
	aVF	0.17 (0.13–0.21)	0.15 (0.1–0.18)	0.0675
	V1	0.09 (0.07–0.1)	0.08 (0.06–0.09)	0.0500
	V2	0.07 (0.06–0.09)	0.08 (0.06–0.09)	0.6826
	V3	0.12 (0.1–0.15)	0.12 (0.09–0.15)	0.3048
	V4	0.12 (0.1–0.14)	0.11 (0.08–0.13)	0.1253
	V5	0.11 (0.08–0.13)	0.09 (0.07–0.11)	0.0531
	V6	0.1 (0.07–0.12)	0.09 (0.06–0.1)	0.0320
Entropy	I	3.1 (3–3.1)	3.1 (3.1–3.1)	0.1403
	II	3.1 (3.1–3.2)	3.1 (3.1–3.2)	0.6330
	III	3.1 (3.0–3.1)	3.1 (3.0–3.1)	0.7376
	aVR	3.1 (3.1–3.2)	3.1 (3.1–3.2)	0.9979
	aVL	3.0 (3.0–3.1)	3.0 (3.0–3.1)	0.5215
	aVF	3.1 (3.1–3.2)	3.1 (3.1–3.2)	0.9139
	V1	3.0 (2.9–3.0)	3.0 (2.9–3.1)	0.9474
	V2	3.0 (3.0–3.1)	3.0 (2.9–3.1)	0.3883
	V3	3.1 (3.0–3.2)	3.1 (3.0–3.1)	0.3251
	V4	3.1 (3.1–3.2)	3.1 (3.1–3.2)	0.2341
	V5	3.1 (3.1–3.2)	3.1 (3.1–3.2)	0.7696
	V6	3.1 (3.1–3.2)	3.1 (3.1–3.2)	0.9558
Sample entropy	I	0.30 (0.21–0.35)	0.29 (0.22–0.31)	0.8638
	II	0.22 (0.19–0.24)	0.25 (0.20–0.28)	0.0369
	III	0.29 (0.21–0.32)	0.34 (0.24–0.42)	0.0284
	aVR	0.22 (0.18–0.24)	0.24 (0.20–0.26)	0.0374
	aVL	0.43 (0.29–0.52)	0.43 (0.31–0.56)	0.6519
	aVF	0.23 (0.19–0.26)	0.27 (0.20–0.32)	0.0430
	V1	0.25 (0.2–0.28)	0.29 (0.24–0.33)	0.0046
	V2	0.35 (0.25–0.4)	0.34 (0.27–0.41)	0.7737
	V3	0.24 (0.19–0.27)	0.25 (0.21–0.28)	0.4489
	V4	0.23 (0.2–0.27)	0.26 (0.21–0.29)	0.0600
	V5	0.23 (0.18–0.24)	0.26 (0.21–0.30)	0.0512
	V6	0.23 (0.18–0.26)	0.26 (0.21–0.30)	0.0109
Number of peaks (*n*)	I	4.9 (1.5–6.2)	3.4 (1.5–3.8)	0.1576
	II	1.6 (1.0–1.7)	1.8 (1.0–2.3)	0.4370
	III	3.1 (1.5–4.2)	3.4 (1.4–5)	0.3320
	aVR	1.6 (1.0–1.8)	1.5 (1.1–1.8)	0.5254
	aVL	6.5 (4.1–8.2)	6.0 (3.6–7.7)	0.5061
	aVF	1.9 (1.0–2.9)	2.2 (1.0–2.9)	0.5297
	V1	3.0 (2.0–3.7)	3.0 (2.0–3.4)	0.9703
	V2	4–2 (2.3–5.2)	3.9 (2.6–4.7)	0.6124
	V3	2.1 (1.0–2.9)	2.0 (1.0–2.5)	0.7981
	V4	1.8 (1.0–2.7)	2.1 (1.0–2.9)	0.1876
	V5	1.9 (1.0–2.2)	2.0 (1–0–2.5)	0.3216
	V6	1.9 (1.0–2.6)	2.1 (1.1–2.6)	0.4038

Data are presented as median (25–75 percentiles). Values in bold refer to statically significant values.

*α* = 0.00076.

### Classifiers evaluation

Classification results are only reported for test set according to the metrics listed in section ‘Performance metrics’.

*Table 4* shows baseline results according to metrics and classifiers. Ensemble models (e.g. Random Forest, Majority Voting, Stacking, Bagging, AdaBoost, and GBoost) showed on average better performances with respect to basic classifiers (e.g. KNN, DT, and SVM). In this preliminary experiment, Bagging on DT proved to be the best-performing algorithm with a weighted F1-score of above 79%.

**Table 4:** Baseline experiment results according to evaluation metrics selected

Overall metrics					
Classifier	Acc. (%)	Sen. (%)	Spec. (%)	MF1 (%)	WF1 (%)
KNN	72.5	84.3	51.3	68.1	71.7
DT	66.3	69.6	60.2	64.2	66.8
SVM	65.4	68.2	60.0	63.4	66.0
Random Forest	78.9	84.4	59.7	75.8	78.3
Majority Voting	72.3	79.9	58.1	69.2	72.1
Stacking	70.7	84.9	44.1	65.3	69.4
Bagging	79.8	83.6	73.6	78.0	79.9
AdaBoost	77.2	93.5	47.0	71.6	75.3
GBoost	79.4	79.4	56.4	75.5	78.4

In order to boost classifiers’ performances, different data balancing techniques were investigated. These included Random Oversampling, Synthetic Minority Oversampling Technique (SMOTE), SVM-SMOTE, Bord-SMOTE, and Adaptive Synthetic (ADASYN). Moreover, weighted class correction for classifier’s loss was tested. Techniques’ effect was evaluated during the training and validation phase, the best-performing framework was then applied to the test set. In *Table 5*, only the best results are reported for each model. KNN, Majority Voting, and Bagging did not benefit from balancing. In contrast, Weighted Class, Adasyn, and SMOTE techniques help to boost the remaining classifiers’ performances. Among these, AdaBoost model reached the highest values for all the considered metrics (Acc. = 81.4%, Sen. = 86.5%, Spec. = 73.8%, MF1 = 79.5%, and WF1 = 81.4%).

**Table 5:** Models results according to different data balancing techniques

Overall metrics						
Classifier	Data balancing	Acc. (%)	Sen. (%)	Spec. (%)	MF1 (%)	WF1 (%)
KNN	—	72.5	84.3	51.3	68.1	71.1
DT	Adasyn	66.3	56.2	85.5	66.1	66.8
SVM	SMOTE	71.6	71.7	73.4	70.4	72.2
Random Forest	SMOTE	78.3	82.4	72.1	76.5	78.4
Majority Voting	—	72.3	79.9	58.1	69.2	72.1
Stacking	SMOTE	79.8	90.2	49.8	78.0	79.9
Bagging	—	79.8	83.6	73.6	78.0	79.9
**AdaBoost**	Weighted class	**81**.**4**	**86**.**5**	**73**.**8**	**79**.**5**	**81**.**4**
GBoost	SMOTE	78.8	81.1	75.4	77.1	78.9

Values in bold refer to statically significant values.

## Discussion

To the best of our knowledge, this is the first study that systematically assessed the performance of AI-based models to identify BrS patients based on P-wave features only. Importantly, the ML algorithms adopted in our analysis were blinded to the underlying ventricular Brugada ECG phenotype. This means that the algorithm adequately identifies Brugada patients based on the atrial phenotype, irrespective of the manifest or concealed Brugada type 1 ECG.

Traditionally the identification of BrS has been based on the evaluation of the depolarization and repolarization phase of the 12-lead ECG, and the Brugada type 1 ECG is currently considered the exclusive diagnostic hallmark of the syndrome.^1,2^ Challenges associated with the ECG diagnosis are related to the intermittent and dynamic features of the ECG pattern of these patients. In the presence of a non-diagnostic pattern, guidelines indicate the administration of sodium channel-blocking drugs to unmask the type 1 ECG. Ajmaline challenge is the most valuable diagnostic tool due to its high sensitivity and specificity.^1^ However, it may expose paediatric patients to an increased risk of ventricular arrhythmias during the test.^16^ Indeed, paediatric BrS family members may be carriers of *SCN5A* variants, be exposed to a risk of ventricular arrhythmias, and have an age-dependent response to the sodium channel blocker test.^16–19^ However, the ideal age for the drug test is controversial.^17^ In these cases, an ECG obtained during a febrile episode is useful to rule out a Brugada type 1 ECG appearance, still it cannot be considered a substitute of the sodium channel blocker test. Furthermore, the drug test should be performed in a safe environment (i.e. intensive care and catheterization laboratory), and this is not possible in many centres due to healthcare facility issues. Therefore, having an AI-based tool to identify patients with BrS patients is of utmost importance as it can avoid drug test and reduce the arrhythmic risk during specific conditions (fever, drugs).

Moreover, in up to 34% of patients with spontaneous BrS, the diagnosis is established after drug challenge by the means of follow-up ECGs or ECG Holter monitoring with high precordial leads that reveal the presence of a spontaneous Brugada type 1 ECG. The identification of this category of patients is of utmost importance, due to the diagnostic and prognostic implication of carrying a spontaneous Brugada type 1 ECG.^20^

The findings of our analysis can be also considered valuable in the diagnostic assessment of BrS, when used in combination with other ventricular parameters that can be found abnormal in Brugada patients (i.e. fragmented QRS, early repolarization pattern, S-Wave in DI, and first-degree atrioventricular [AV] block).

Previous studies on long-QT syndrome using ML methods have shown to improve the diagnosis or even identify concealed forms of long-QT syndrome (LQTS).^13,14^ Recently, Liu *et al.* introduced the first deep neural network for Brugada syndrome recognition, training it to recognize the right bundle branch block pattern and then diagnose Brugada type I. A total of 2257 right bundle branch block and 276 BrS instances of 10 s 12-lead ECG served as input to the network. The model achieved notable precision, with an area under the curve (AUC) of 0.89.^21^ Liao *et al.* developed a DL system to continuously monitor Brugada type 1 pattern in 24 h ambulatory 12-lead ECGs, training it on 1190 12-lead ECGs and 380 12-lead Holter ECGs. This resulted in a remarkable AUC of 0.98 for classifying Brugada type 1 patterns from both standard and Holter 12-lead ECGs.^22^

Vozzi *et al.* presented a novel approach using a recurrent neural network on data from four consecutive beats and three leads (V1, V2, V3) to diagnose Brugada type 1 ECG on a cohort of 156 patients. The model achieved a good accuracy if trained with V2 only (80.2%).^12,23^

Similarly, our study included 123 patients from which 2228 averaged P-waves were extracted. Compared with previous studies, our model achieved similar performance, with an accuracy of 81.4%. Remarkably, this achievement was attained through the exclusive utilization of the atrial signal for training, representing a novel advancement in the field.

### Artificial intelligence models to detect the concealed atrial phenotype of Brugada syndrome

The susceptibility to the development of atrial arrhythmias and AF reflects the involvement of the atrial myocardium in the manifestation of the syndrome.^3,4^ Recent studies have reported the presence in BrS patients of an altered atrial phenotype characterized by abnormal P-wave parameters, despite the absence of AF history, and of an atrioventricular ECG phenotypic mismatch, being P-waves abnormalities detected even in the absence of an overt Brugada type 1 ECG.^6^ Moreover, prolonged atrial conduction time at ECG imaging has been recently reported.^8^ Gene-related atrial cardiomyocytes alterations could create favourable structural and functional substrates for the genesis of re-entry circuits underlying atrial arrhythmias. These observations open the way to new diagnostic methods for BrS identification, no longer based only on the study of ventricular abnormalities but also on the study of ECG-visible atrial abnormalities.

Artificial intelligence models based on P-wave features have been already used to identify patients prone to AF.^10,11^ In a study by Yang *et al.*,^11^ an algorithm to quantify temporal and spatial alterations of the P-wave, the ML models achieved an AUC of 0.64, showing that ML performed better compared to DL in a limited series of patients. In another study, an AI-enabled network had the ability to predict AF from a sinus rhythm single-lead ECG.^10^ In our study, performed on subjects without any history of AF, the investigation of the characteristics associated with P-waves in the BrS group compared to the control group revealed the presence of abnormalities. These primarily concerned the duration and the area of P-wave, as reported in previous studies.^6,24^ The presence of statistically significant differences led to the consideration that ML models can distinguish BrS patients from a control group based on P-wave characteristics only. The results obtained in the baseline experiment highlighted the influence of classifiers on the imbalance of the initial database, consisting of 79 subjects with BrS and 44 healthy subjects. To address this issue and improve model performance, various data balancing techniques were investigated. For most models, these techniques proved beneficial for classification purposes. Indeed, the best result achieved was associated with the AdaBoost classifier that, through weighted class loss, achieved an accuracy of 81.4%, a sensitivity of 86.5% and a specificity of 73.8%. Moreover, the employment of ensemble techniques led to improved performance in contrast to the fundamental classifiers, such as KNN and DT. However, it is important to remark that this performance boost is associated to an escalation in model complexity. AdaBoost, in particular, relied on an ensemble of 50 decision trees, displaying the trade-off between model complexity and predictive power.

The objective evaluation of the obtained result is challenging given the novelty of the study and the absence in the literature of similar studies. Nevertheless, our results demonstrate the possibility of conducting the diagnosis of BrS in an alternative manner providing valuable support to clinicians, especially in ambiguous conditions such as in case of type 2 ECGs and non-interpretable ventricular patterns (patients with pacemakers or left bundle branch block). However, future studies are needed to confirm if AI models can be clinically used to aid cardiologists in identifying BrS without the need of a provocative drug challenge.

### Limitations

Our study has certain limitations. It is a retrospective study conducted, due to the rarity of the condition, in a small population of adult patients with heterogeneous clinical characteristics. Brugada patients were 10 years older than controls, and age is known to influence P-wave features.^25^ In order to reduce the impact of this potential confounders, after dividing training set and test set, we ensured that test set contained only age- and sex-matched patients among the two groups to minimize any potential bias related to age differences.

From a technical perspective, the difficulty of the classification task and the use of simple feature-based models certainly make the performance less competitive compared to other classifiers used in different contexts. In the future, it will be valuable to increase the size and heterogeneity of clinical and ECG data in order to test the robustness of the models and enhance their predictive capability. The models should be tested in paediatric patients with BrS.

## Conclusions

An AI machine-learning model is able to identify patients with BrS based only on P-wave features. These findings confirm the presence of an atrial hallmark and open new horizons for AI-guided BrS diagnosis.

## Supplementary Material

euad334_Supplementary_Data

## Data Availability

The data underlying this article will be shared on reasonable request to the corresponding author.
