# Supplementary material for: Identification of Brugada syndrome based on P-wave features: an artificial intelligence-based approach
Source: Europace. 2023 Nov 7;25(11):euad334. doi: 10.1093/europace/euad334 (PMC10683037; doi:10.1093/europace/euad334)
Supplement: euad334_Supplementary_Data [file euad334_supplementary_data.docx]

***Supplementary material***

**Table 1.** Test set population characteristics.

|  | **Brugada patients**  **(N=21)** | **Negative Ajmaline subjects**  **(N=7)** |
| --- | --- | --- |
| ***Clinical characteristics***  Male sex, n (%)  Age (years)  Family history of SCD, n (%)  Syncope, n (%)  Previous sustained VAs, n (%)  Previous ICD implantation, n (%)  Heart Rate (bpm) | 14 (66.7%)  40±15  2 (9,5%)  4 (19.0%)  1 (4.7%)  6 (28.5%)  79 ± 12 | 5 (71.4%)  39±12  0 (0%)  1 (14.3%)  -  -  75 ± 11 |
| ***Echocardiographic parameters***  LVEF (%)  LA Diameter (mm)  LAVI (ml/m^2^)  RAVI (ml/m^2^) | 61*±*3.5  31±6.1  26.8±4.6  22.6±4.1 | 60.8±2.9  28.4±5.3  24.3±1.5  19.7±3.8 |
